# Leukotoxin of *Bibersteinia trehalosi* Contains a Unique Neutralizing Epitope, and a Non-Neutralizing Epitope Shared with *Mannheimia haemolytica* Leukotoxin

**DOI:** 10.3390/toxins10060220

**Published:** 2018-05-30

**Authors:** Arumugam Murugananthan, Sudarvili Shanthalingam, Sai Arun Batra, Sitara Alahan, Subramaniam Srikumaran

**Affiliations:** 1 Department of Veterinary Microbiology and Pathology, College of Veterinary Medicine, Washington State University, Pullman, WA 99164-7040, USA; ananthan10@gmail.com (A.M.); sudar@vetmed.wsu.edu (S.S.); sai@vetmed.wsu.edu (S.A.B.); sitara.alahan@wsu.edu (S.A.); 2 Department of Parasitology, Faculty of Medicine, University of Jaffna, Jaffna, Sri Lanka

**Keywords:** *Bibersteinia trehalosi*, *Mannheimia haemolytica*, leukotoxin, neutralizing epitope

## Abstract

*Bibersteinia trehalosi* and *Mannheimia haemolytica*, originally classified as *Pasteurella haemolytica* biotype T and biotype A, respectively, under Genus *Pasteurella* has now been placed under two different Genera, *Bibersteinia* and *Mannheimia*, based on DNA-DNA hybridization and 16S RNA studies. While *M. haemolytica* has been the predominant pathogen of pneumonia in ruminants, *B. trehalosi* is emerging as an important pathogen of ruminant pneumonia. Leukotoxin is the critical virulence factor of these two pathogens. While the leukotoxin of *M. haemolytica* has been well studied, the characterization of *B. trehalosi* leukotoxin has lagged behind. As the first step towards addressing this problem, we developed monoclonal antibodies (mAbs) against *B. trehalosi* leukotoxin and used them to characterize the leukotoxin epitopes. Two mAbs that recognized sequential epitopes on the leukotoxin were developed. One of them, AM113, neutralized *B. trehalosi* leukotoxin while the other, AM321, did not. The mAb AM113 revealed the existence of a neutralizing epitope on *B. trehalosi* leukotoxin that is not present on *M*. *haemolytica* leukotoxin. A previously developed mAb, MM601, revealed the presence of a neutralizing epitope on *M. haemolytica* leukotoxin that is not present on *B. trehalosi* leukotoxin. The mAb AM321 recognized a non-neutralizing epitope shared by the leukotoxins of *B. trehalosi* and *M. haemolytica*. The mAb AM113 should pave the way for mapping the leukotoxin-neutralizing epitope on *B. trehalosi* leukotoxin and the development of subunit vaccines and/or virus-vectored vaccines against this economically important respiratory pathogen of ruminants.

## 1. Introduction

**Figure 1:**
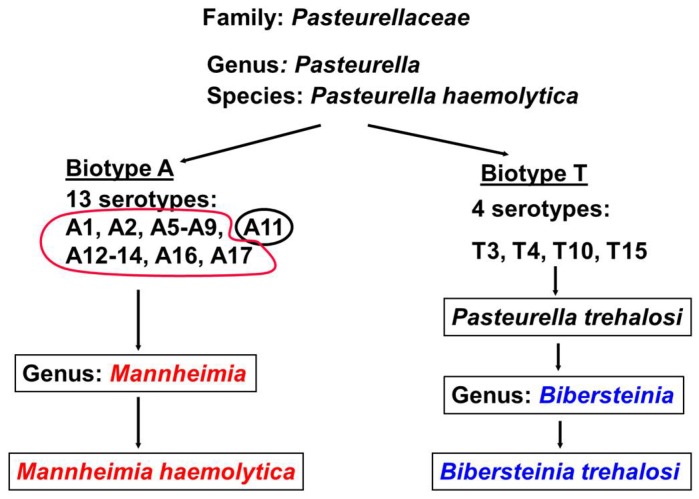
Previous and current taxonomical classification of *Bibersteinia trehalosi* and *Mannheimia haemolytica.*

*Mannheimia haemolytica* and *Bibersteinia trehalosi* are important pathogens of pneumonia in domestic and wild ruminants worldwide [1,2,3]. *M. haemolytica* and *B. trehalosi* were originally known as *Pasteurella haemolytica* biotype A and *Pasteurella haemolytica* biotype T of species *Pasteurella haemolytica* under Genus *Pasteurella* in the Family *Pasteurellaceae* (Figure 1). *Pasteurella haemolytica* biotype A had 13 serotypes and *Pasteurella haemolytica* biotype T had 4 serotypes. Subsequently, in 1999, based on the results from DNA-DNA hybridization and 16S RNA studies, all serotypes of *P. haemoltica* biotype A were grouped under a newly created Genus *Mannheimia* [4]. All serotypes became *Mannheimia haemolytica* except A11 which became *Mannheimia glucosida*. All four serotypes of *Pasteurella haemolytica* biotype T were named as *Pasteurella trehalosi* under Genus *Pasteurella*. Further taxonomical analysis in 2007 resulted in the creation of a new Genus, *Bibersteinia*, under which all four *Pasteurella trehalosi* serotypes were placed as *Bibersteinia trehalosi* [5].

While *M. haemolytica* has been the predominant pathogen of pneumonia in ruminants, *B. trehalosi* is emerging as an important pathogen of ruminant pneumonia [6,7,8]. In conjunction with active viral infection and stress factors, *M. haemolytica* and *B. trehalosi* cause an acute fibrinonecrotic pleuropneumonia [9]. Both these organisms produce several virulence factors including the capsule, outer membrane proteins, adhesins, neuraminidase, lipopolysaccharide and leukotoxin (Lkt) [10]. Lkt is a 102 kD protein made up of 953 amino acids. Based on the observation that Lkt-deletion mutants cause no mortality [11] or reduced mortality and milder lung lesions [12,13] than the wild-type organisms, Lkt is accepted as the most important virulence factor of these organisms. Lkt belongs to the family of RTX (repeats in toxins) toxins and shares extensive homology with the exotoxins produced by *Escherichia coli* [14], *Actinobacillus pleuropneumoniae* [15], and *Actinobacillus actinomycetemcomitans* [16]. However, cytolytic activity of Lkt is specific for ruminant leukocytes [17,18]. Although all subsets of leukocytes are susceptible to the cytolytic effects of Lkt, polymorphonuclear cells (PMNs) are the most susceptible subset [19]. Lkt-induced PMN lysis and degranulation are the primary causes of acute inflammation and lung injury characteristic of this disease [20,21,22].

Lkt-neutralizing antibodies offer protection against challenge with wild-type *M. haemolytica* [23,24]. Hence vaccines against this organism contain Lkt as the primary component [25,26]. While the Lkt of *M. haemolytica* has been studied extensively [22,27,28,29], characterization of *B. trehalosi* Lkt has lagged behind. As the first step towards addressing this problem, we developed monoclonal antibodies against the Lkt of *B. trehalosi* and used them to characterize the Lkt epitopes. Although all serotypes of *B. trehalosi* can cause respiratory disease in ruminants, serotype T10 has been commonly isolated from pneumonic lungs of sheep and cattle. Likewise, while all serotypes of *M. haemolytica* can cause respiratory disease in ruminants, serotype A1 predominantly causes pneumonia in cattle, while serotype A2 is the common cause of pneumonia in domestic and wild sheep [21]. Therefore, in this study we focused on the Lkts of *B. trehalosi* serotype T10 and *M. haemolytica* serotype A1 and serotype A2. 

## 2. Results and Discussion

### 2.1. Monoclonal Antibody AM113 Reacts Only with B. trehalosi Lkt While AM 321 Reacts with Lkts of B. trehalosi and M. haemolytica

A total of 304 hybridoma clones were obtained from two independent fusions. To simplify the screening process, culture fluids from these clones were first tested by ELISA on plates coated with a mixture of Lkts of *B. trehalosi* and *M. haemolytica*. The clones that were positive by this ELISA were subsequently tested by another ELISA on plates coated with Lkt of *B. trehalosi* or *M. haemolytica* serotype A1 or serotype A2. These two ELISAs identified two positive clones. One of them secreted a mAb (AM113) that reacted only with the Lkt of *B. trehalosi* while the other one (AM321) reacted with Lkts of *B. trehalosi* and *M. haemolytica* serotypes A1 and A2 (data not shown). 

### 2.2. Western Blot Analysis Confirms the Specificity of mAbs AM113 and AM321

**Figure 2:**
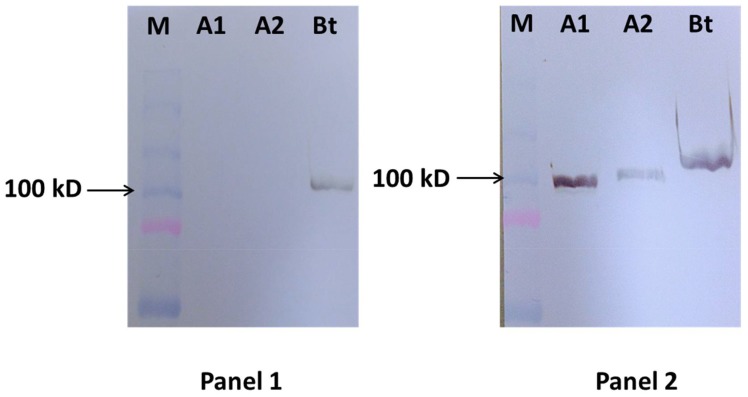
Western blot analysis confirms the specificity of mAbs AM113 and AM321. Lkts of *B. trehalosi* and *M. haemolytica* serotypes A1 and A2 were subjected to SDS-PAGE followed by western blot analysis with culture fluids from the hybridomas AM113 (Panel 1) and AM321 (Panel 2). Lanes M, A1, A2, and Bt represent the molecular weight standards, Lkts of *M. haemolytica* serotype A1, serotype A2, and *B. trehalosi*, respectively.

Western blot analysis of mAbs AM113 and AM321 with Lkts of *B. trehalosi* and *M. haemolytica* revealed that mAb AM113 reacts only with *B. trehalosi* Lkt, while AM321 reacts with Lkts of *B. trehalosi* and *M. haemolytica* serotypes A1 and A2, confirming the results of ELISA (Figure 2). Both mAbs reacted with an approximately 100 kD band which is consistent with the molecular weight of Lkt. It is apparent that the Lkt bands of *B. trehalosi* and *M. haemolytica* serotypes A1 and A2 are not identical in size, which is likely because the Lkt is not a very stable protein. Under conditions used in SDS-PAGE, it tends to break down into smaller components which has been reported in earlier studies as well [27].

Since SDS-PAGE, the first step in western blot analysis, denatures the Lkt protein, reactivity of the mAbs with the Lkts in the western blot analysis indicated that both mAbs AM113 and AM321 recognize sequential epitopes on the Lkt. Taken together, the results of ELISA and western blot assay indicated that mAb AM113 recognizes a sequential epitope unique to *B. trehalosi* Lkt, while mAb AM321 recognizes a sequential epitope that is shared among the Lkts of *B. trehalosi* and *M. haemolytica* serotypes A1 and A2.

### 2.3. Monoclonal Antibody AM113 Neutralizes B. trehalosi Lkt While mAb AM321 Does Not

**Figure 3:**
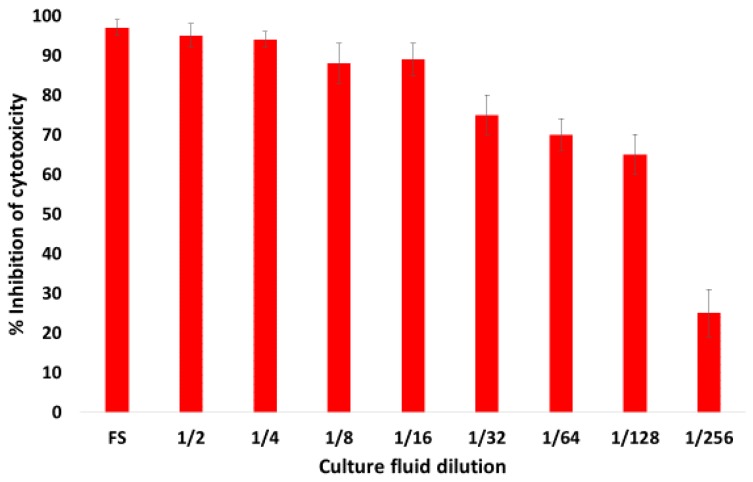
Lkt neutralizing activity of mAb AM113. Serial dilutions of mAb AM113 in the form of culture fluid were incubated with *B. trehalosi* Lkt dilution that gives 50% cytotoxicity. Following incubation, the target cells were added to the Lkt-mAb mixture and the cytotoxicity assay was completed. Culture fluid containing the mAb AM113 exhibited a neutralizing antibody titer of 1:128. Error bars indicate standard deviations of the means.

The cytotoxicity inhibition assay revealed that mAb AM113 neutralizes *B. trehalosi* leukoxin (Figure 3). The neutralizing titer of culture fluid containing mAb AM113 was 1:128. The mAb AM321 did not exhibit leukotoxin-neutralizing activity (data not shown).

### 2.4. Monoclonal Antibodies Reveal the Presence of Unique Neutralizing Epitopes on the Lkts of B. trehalosi and M. haemolytica Serotype A1

We used the mAb AM113 developed in this study along with another anti-Lkt mAb, MM601, developed previously by our laboratory [27], in the cytotoxicity inhibition assay, to analyze the presence of neutralizing epitopes on the Lkts of *B. trehalosi* and *M. haemolytica*. The mAb MM601 neutralizes *M. haemolytica* serotype A1 [27]. However, it was not tested against the Lkts of *B. trehalosi* or *M. haemolytica* serotype A2. In the cytotoxicity inhibition assay, the mAb AM113 neutralized the Lkt of *B. trehalosi*, but not the Lkts of *M. haemolytica* serotype A1 or A2. The mAb MM601 neutralized the Lkt of *M. haemolytica* serotype A1, but not the Lkts of *M. haemolytica* serotype A2 or *B. trehalosi* (Table 1). These results indicated that the mAb AM113 recognizes a neutralizing epitope present exclusively on *B. trehalosi* Lkt, while the mAb MM601 recognizes a neutralizing epitope present exclusively on *M. haemolytica* serotype A1 Lkt. 

It is noteworthy that the mAbs AM113 and MM601 do not neutralize *M. haemolytica* serotype A2 Lkt. It is plausible that the neutralizing epitope recognized by mAb AM113 on *B. trehalosi* Lkt, and the neutralizing epitope recognized by mAb MM601 on *M. haemolytica* serotype A1 Lkt, are not present on, or not critical for the cytotoxicity of, *M. haemolytica* serotype A2 Lkt. Previously, Gerbig et al. [28] reported a Lkt-neutralizing mAb that reacts with Lkts of *M. haemolytica* serotypes A1, A5, A6, A7, A8, A9 and A12, but not serotype A2 or *B. trehalosi*. It is tempting to speculate that *M. haemolytica* serotype A2 is antigenically very different from the other serotypes of *M. haemolytica* and *B. trehalosi*. It is interesting to note here that *M. haemolytica* serotype A2 predominantly infects sheep while the other serotypes mostly infect cattle. In an earlier study with serotype-specific polyclonal rabbit antisera, Shewen and Wilkie [30] implied antigenic difference in the Lkt, among the serotypes of *M. haemolytica*. Our study confirms their observation. 

Development of a mAb that neutralizes *B. trehalosi* Lkt is of broad significance. The neutralizing epitope on *M. haemolytica* Lkt recognized by the mAb MM601 has been mapped to amino acids 841 to 872 [31]. Our mAb AM113 should pave the way for mapping a neutralizing epitope on *B. trehalosi* Lkt. Recombinant chimeric proteins containing a specific domain of Lkt that contains a neutralizing epitope, and the outer membrane protein PlpE of *M. haemolytica* have been used as immunogens to induce protective immunity against this organism [32,33]. Mapping of a neutralizing epitope on *B. trehalosi* Lkt and inclusion of the Lkt domain containing the neutralizing epitope in the chimeric protein should broaden the scope of this approach. A single chimeric protein could be developed for use as immunogen against both *M. haemolytica* and *B. trehalosi*. The reactivity of our non-neutralizing mAb AM321 with the Lkts of *B. trehalosi* and *M. haemolytica* serotypes A1 and A2 indicates the presence of epitopes that are shared by these three Lkts. This finding suggests that neutralizing epitopes that are shared by the Lkts of *B. trehalosi* and *M. haemolytica* serotypes A1 and A2 are also likely to exist. Development of mAbs that recognize such epitopes and their inclusion in the chimeric protein should further broaden the utility of this approach. Viral vectors engineered to carry bacterial immunogens have been used as live vaccines [34]. In this regard, attempts have been made to use bovine herpesvirus 1 as a vector to carry Lkt-neutralizing epitope and immunogenic domain of an outer membrane protein of *M. haemolytica* [35]. The mAbs developed in this study should enhance the success of such virus-vectored vaccines.

## 3. Conclusions

1. Lkts of *B. trehalosi* and *M. haemolytica* serotype A1 carry neutralizing epitopes uniquely present on them.
2. Lkts of *B. trehalosi, M. haemolytica* serotype A1, and serotype A2 carry a shared non-neutralizing epitope.
3. The mAb developed in this study should pave the way for mapping of a neutralizing epitope on *B. trehalosi* Lkt, which in turn should help in the development of subunit vaccines and virus-vectored live vaccines against this economically important respiratory pathogen of domestic and wild ruminants.

## 4. Materials and Methods

### 4.1. Production of Lkt

Lkt from *B. trehalosi and M. haemolytica* was produced as described previously [27]. Briefly, bacteria were grown to logarithmic phase in BHI broth (Remel, Lenexa, KS, USA) at 37 °C, harvested by centrifugation (13,500× *g* for 20 min at 4 °C), and resuspended in twice the original culture volume of colorless RPMI 1640 medium (Invitrogen, Carlsbad, CA, USA). After an additional 1–1.5 h of growth at 37 °C, the bacteria were pelleted by centrifugation, and the supernatant fluid was filter-sterilized using a 0.22 μm filter. This supernatant preparation containing Lkt was stored at −20 °C until needed.

### 4.2. Immunization of Mice and Development of Hybridomas

A 100-fold concentrate of the Lkt preparation was used to immunize four eight week-old female BALB/c mice. A mixture of Lkts from *B. trehalosi* and *M. haemolytica* serotype A1 and A2 was used to immunize all four mice. The production and cloning of the hybridomas were performed according to previously published methods [27]. Culture fluids from wells containing visible hybridoma clones were screened for their reactivity to Lkt by an indirect ELISA. Hybridomas from positive wells were sub-cloned three times each, by the limiting dilution method.

### 4.3. ELISA

Presence of antibodies in hybridoma culture fluids was determined by an indirect ELISA described previously [26]. To simplify the screening process, culture fluids from wells containing visible hybridoma clones were first tested by ELISA on plates coated with a mixture of Lkts containing approximately same concentration of *B. trehalosi* and *M. haemolytica* serotype A1 and A2 Lkts. The clones that were positive by this ELISA were subsequently tested by ELISA on plates coated with Lkt of *B. trehalosi* or *M. haemolytica* serotype A1 or serotype A2. Lkt-bound mAbs were detected by the addition of HRP-conjugated goat anti-mouse Ig antibodies followed by the substrate ABTS.

### 4.4. Western Blot Assay

Western blot analysis of the reactivity of mAbs with the Lkts was performed as described previously [27]. Lkt was subjected to SDS-PAGE in 10% gels under reducing conditions, followed by western blot analysis with mAbs in the form of undiluted hybridoma culture fluids. Binding of the mAb to the Lkt was detected by treatment with HRP-conjugated goat anti-mouse Ig antibodies. Visualization of the bands was accomplished with the substrate 4CN.

### 4.5. Cytotoxicity Assay for Detection of Cytotoxicity of Lkt Preparations and Cytotoxicity Inhibition Assay for Detection of Lkt-Neutralizing Activity of mAbs

Cytotoxicity of Lkt preparations was determined by using the previously described MTT (3-[4-dimethylthiazoyl-2-yl]-2,5-diphenyl tetrazolium bromide) [Sigma Chemical Co., St. Louis, MO, USA]) dye-reduction cytotoxicity assay [27]. This assay measures the ability of the endoplasmic reticulum-resident enzymes in viable cells to convert the tetrazolium dye into a purple formazan precipitate which is dissolved in cold acid isopropanol. Color intensity is directly proportional to the viability of the cells. The bovine lymphoma cell-line BL-3 was used as target cells. Briefly, BL-3 cells were re-suspended in colorless RPMI 1640 (Invitrogen, Carlsbad, CA, USA) at a concentration of 5 × 10^6^ cells mL^−1^ and seeded into 96 well round bottom microtiter plates (50 uL/well) containing the serially diluted Lkt preparation (50 uL/well) in duplicates and incubated at 37 °C for 1 h. Cells were centrifuged at 600× *g* for 5 min following incubation, and the supernatant fluid was discarded. The cell pellets were re-suspended in 100 µL of colorless RPMI 1640, and 20 µL of 0.5% MTT dye were added to each well. After 1 h of incubation at 37 °C, the plates were centrifuged at 600× *g* for 5 min and the supernatant fluid was removed. The formazan precipitate was dissolved in 100 µL acid isopropanol and the optical density (OD) of the samples was measured using an ELISA reader at 540 nm. The percent cytotoxicity was calculated as follows: % cytotoxicity = [1 − (OD of toxin-treated cells/OD of toxin-untreated cells)] × 100. 

Lkt-neutralizing activity of the mAbs in the culture fluid was determined by the MTT dye-reduction cytotoxicity assay described above, with the modification that the Lkt was pre-incubated with serially diluted culture fluid samples for 1 h at 4 °C before the addition of the cells. In this assay, the Lkt was used at a dilution which caused 50% cytotoxicity to BL-3 cells. The rest of the assay was identical to that described above. Percent inhibition was calculated as described previously [27] and outlined below. % inhibition = [1 − (% cytotoxicity in the presence of mAb/% cytotoxicity in the absence of mAb)] × 100. The highest dilution of the mAb that caused at least 50% inhibition of Lkt-induced cytotoxicity was considered as the titer. 

## Figures and Tables

**Table 1:** Leukotoxin-neutralizing activity of mAbs AM113 and MM601. Leukotoxin-neutralizing activity of mAbs AM113 and MM601 was detected by the cytotoxicity inhibition assay of culture fluids containing the mAbs, with the leukotoxins of *B. trehalosi*, *M. haemolytica* serotype A1 and serotype A2. BL-3 cells were used as the target cells.

*Monoclonal Antibody*	Leukotoxin Neutralization		
	*B. trehalosi*	*M. haemolytica* A1	*M. haemolytica* A2
**AM113**	+	-	-
**MM601**	-	+	-
